# Correction: Shi et al. A Comprehensive Overview of the Antibiotics Approved in the Last Two Decades: Retrospects and Prospects. *Molecules* 2023, *28*, 1762

**DOI:** 10.3390/molecules30020209

**Published:** 2025-01-07

**Authors:** Zhenfeng Shi, Jie Zhang, Lei Tian, Liang Xin, Chengyuan Liang, Xiaodong Ren, Min Li

**Affiliations:** 1 Department of Urology Surgery Center, Xinjiang Uyghur People’s Hospital, Urumqi 830002, China; 2 Faculty of Pharmacy, Shaanxi University of Science & Technology, Xi’an 710021, China; 3 Medical College, Guizhou University, Guiyang 550025, China; 4 College of Pharmacy, Xinjiang Medical University, Urumqi 830054, China

In the original publication [1], some citation errors occurred due to issues with our reference management. During the manuscript preparation, we revised several citations in the main text, but the reference list was not updated accordingly. This led to mismatches between the in-text citations and the reference list. To address this issue, we have updated the reference list to ensure it accurately corresponds with the in-text citations.

With this correction, the order of some references has been adjusted accordingly. The authors state that the scientific conclusions are unaffected. This correction was approved by the Academic Editor. The original publication has also been updated.
